# Urine kidney injury molecule-1 predicts subclinical kidney disease among persons living with HIV initiating tenofovir disoproxil fumarate-based ART in Zambia

**DOI:** 10.3389/fneph.2024.1468409

**Published:** 2025-01-06

**Authors:** Freeman W. Chabala, Edward D. Siew, C. William Wester, Alana T. Brennan, Masauso M. Phiri, Michael J. Vinikoor, Sepiso K. Masenga, Muktar H. Aliyu

**Affiliations:** ^1^ The Institute of Basic and Biomedical Sciences, Levy Mwanawasa Medical University, Lusaka, Zambia; ^2^ Department of Nephrology, Nephrology Vanderbilt O’Brien Center for Kidney Disease, Nashville, TN, United States; ^3^ Tennessee Valley Health Systems (TVHS), Veterans Affairs, Nashville, TN, United States; ^4^ University of Alabama at Birmingham, Birmingham, AL, United States; ^5^ Department of Nephrology, Nephrology Vanderbilt Institute for Global Health (VIGH), Nashville, TN, United States; ^6^ Department of Global Health, Boston University School of Public Health, Boston, MA, United States; ^7^ Health Economics and Epidemiology Research Office, Faculty of Health Sciences, University of the Witwatersrand, Johannesburg, South Africa; ^8^ Department of Epidemiology, Boston University School of Public Health, Boston, MA, United States; ^9^ Center for Primary Care Research, School of Medicine, University of Zambia, Lusaka, Zambia; ^10^ Department of Internal Medicine, University of Zambia, Lusaka, Zambia; ^11^ Department of Physiological Sciences, Mulungushi University School of Medicine and Health Sciences, Livingstone, Zambia; ^12^ Department of Health Policy, Vanderbilt University Medical Center (VUMC), Nashville, TN, United States; ^13^ Department of Medicine, Vanderbilt University Medical Center (VUMC), Nashville, TN, United States

**Keywords:** kidney injury molecule-1, neutrophil gelatinase-associated lipocalin, acute kidney injury, HIV, tenofovir disoproxil fumarate-associated nephrotoxicity, Zambia

## Abstract

**Introduction:**

Antiretroviral therapy (ART) increases the life expectancy of persons living with HIV (PLWH), but not without potentially serious adverse effects. Tenofovir disoproxil fumarate (TDF) can cause nephrotoxicity, manifesting as acute kidney injury (AKI) that may persist after treatment discontinuation. Kidney injury biomarkers such as kidney injury molecule-1 (KIM-1), retinol-binding protein-4 (RBP-4), interleukin-18 (IL-18), and neutrophil gelatinase-associated lipocalin (NGAL) can aid early diagnosis and predict TDF-associated nephrotoxicity. This study aimed to determine whether the change from baseline in urine KIM-1 (δKIM-1) and NGAL (δNGAL) following 2 weeks of TDF use could predict subclinical TDF-associated nephrotoxicity before the overt manifestation as acute kidney disease after 3 months.

**Methods:**

A prospective cohort study of 205 PLWH was conducted at the Adult Center for Infectious Disease Research (AIDC) in Lusaka, Zambia. ART-naïve PLWH who were starting treatment with TDF with intact kidney function [estimated glomerular filtration rate (eGFR)> 60 mL/min/1.73m^2^] were followed at initiation, 2 weeks, and approximately 3 months to determine the incidence of TDF-associated nephrotoxicity. We measured urine KIM-1 and NGAL at baseline and after 2 weeks of treatment to determine if it predicted subclinical nephrotoxicity. The presence of TDF-associated nephrotoxicity was defined according to the established acute kidney disease and disorders criteria (AKD) as having either 1) one or more episodes of eGFR< 60ml/min/1.73m^2^ within 3 months, 2) a reduction in eGFR of greater than 35% (from baseline) within 3 months, and/or 3) an increase in serum creatinine of more than 50% (from baseline) within 3 months.

**Results:**

The incidence of TDF-associated nephrotoxicity was 22%. Baseline eGFR, creatinine, age, female sex, and BMI predicted the risk of overt TDF-associated nephrotoxicity. The median baseline KIM-1-to-creatinine and NGAL-1-to-creatinine ratios of the participants who developed overt TDF-associated nephrotoxicity and those who did not were not significantly different. However, every 1 pg/mg increase in δKIM-1 was associated with a 41% higher risk of TDF-associated nephrotoxicity. No association was observed with δNGAL.

**Conclusions:**

The incidence of TDF-associated nephrotoxicity was high. Change in KIM-1 level within 2 weeks of the initiation of TDF treatment predicted subclinical TDF-associated nephrotoxicity before overt manifestation as acute kidney disease while δNGAL within the same period did not predict subclinical TDF-associated nephrotoxicity.

## 1 Introduction

Sub-Saharan Africa (SSA) remains the epicenter of the HIV/AIDS pandemic, with more than 70% of the global HIV burden and 52% of HIV-related deaths (1). The widescale implementation of antiretroviral therapy (ART) has ameliorated the impact of HIV-associated morbidity and mortality, but not without ART-associated adverse drug events, some of which can be serious (2–4). Tenofovir disoproxil fumarate (TDF), a leading ART agent that is increasingly used for HIV prevention and chronic hepatitis B virus treatment, can uncommonly lead to nephrotoxicity that may manifest as acute kidney injury (AKI) associated with proximal tubular damage and can progress to chronic kidney disease (CKD) and/or end-stage kidney disease (ESKD) requiring dialysis (5, 6). AKI causes significant morbidity and mortality (7); it is a risk factor for a myriad of complications, including CKD, ESKD and cardiovascular disease (8, 9). The risk of these complications remains high, even following treatment discontinuation or dose modification of the causative agent (8–12). In SSA, the outcomes of ESKD are very poor; therefore, preventing TDF-related complications is of paramount importance.

TDF causes proximal tubular damage by drug-transporter interactions; specifically, the drug accumulates and inhibits mitochondrial DNA (mDNA) polymerase-γ, electron transport chain (ETC) complexes, depletes mitochondria, and reduces cell survival (13–16). Unfortunately, monitoring of kidney health entails measuring serum creatinine levels, which often detects kidney injury late in its course, often at a late stage when the damage is irreversible (17). Before creatinine levels rise, several blood and urine biomarkers may play a role in recognizing early kidney tubular injury, including interleukin-18 (IL-18), kidney injury molecule-1 (KIM-1), neutrophil gelatinase-associated lipocalin (NGAL) (18–21), N-acetyl-β-D-glycosaminidase (NAG) (22), liver-type fatty acid-binding protein (L-FABP) (23–25), retinol-binding protein-4 (RBP-4), and β2-microglobulin (26). This study assessed the value of urine KIM-1 and NGAL for the early detection of TDF-associated nephrotoxicity because the two biomarkers are produced in the proximal renal tubular cells which TDF nephrotoxicity primarily affects. For example, the blood/urine concentration of KIM1, a type 1 transmembrane protein of 60-90 KD molecular weight with an immunoglobulin and mucin domain, increases following kidney tubular injury (27). NGAL, in turn, is expressed in proximal tubular cells as a 135 KD barrel-shaped gelatinase heterodimer of the lipocalin family with the ability to bind lipophilic molecules and be sensitive to kidney injury (28).

There is a paucity of knowledge about the diagnostic value of KIM-1 and NGAL in detecting subclinical nephrotoxicity among Africans living with HIV within the early stages of treatment with TDF before overt manifestation as acute kidney disease and this study aims to close that knowledge gap. We hypothesized that the change in urine KIM-1 and urine NGAL levels can indicate subclinical nephrotoxicity after 2 weeks of exposure to TDF, since these two biomarkers are highly expressed in the proximal tubular cells, the primary site for TDF-associated kidney injury.

## 2 Methods

This was a prospective observational cohort study of 205 persons living with HIV (PLWH) who were recruited at the start of their TDF-based therapy and followed for a maximum period of 4 months at the Adult Infectious Disease Research Centre (AIDC), Zambia’s largest HIV referral and treatment center. Participants were ART-naïve adults (18+ years) who were initiating TDF-based ART under standard treatment conditions and had intact/preserved kidney function at enrollment [estimated glomerular filtration rate (eGFR)≥ 60 mL/min/1.73m^2^ based on serum creatinine with eGFR calculated using the Chronic Kidney Disease Epidemiology Collaboration (CKD-EPI) equation]. Clinicians initiated the participants’ treatment and treated them according to the Zambian National ART treatment guidelines (24) and their expert clinical discretion to minimize the risk of kidney injury. In addition to 300mg TDF, ART contained 300mg lamivudine (3TC) and 600mg efavirenz (EFV) in a fixed-dose combination pill taken once daily. After ART was started, eGFR was re-assessed at 2 weeks and 3 months. None of the participants were reported to have been taking any specific nephrotoxic drugs.

The primary outcome was the incidence of TDF-associated nephrotoxicity after 3 months of TDF-based ART defined according to the acute kidney disease and disorders (AKD) criteria (29) as having either 1) eGFR< 60ml/min/1.73m^2^ at 3 months, 2) a reduction in eGFR of greater than 35% within 3 months, or 3) increase in serum creatinine of more than 50% from baseline at 3 months. In this study, participants were lost to follow-up (LTFU) if they did not attend their scheduled 3-month visit and were not traced during contact tracing. Contact tracing involved contacting the participants using the contact number they provided. If they were unreachable after four attempts weekly in the fourth month of follow-up and no kidney function data was found for the third visit in their electronic health record (EHR), they were deemed to be LTFU.

### 2.1 Participant recruitment

Participants were recruited and followed up starting on 24 September 2018 and ending on 16 September 2019. A REDCap (30) questionnaire was used in the interviews to collect baseline demographics and the participants’ medical history. The interview collected information on their history of concomitant traditional medicine/herb use, non-steroidal autoinflammatory drug (NSAID) use, lifestyle choices such as smoking and alcohol use, and history of comorbidities. Comorbidities included a known history of diabetes, hypertension, chronic hepatitis B or C, and/or tuberculosis, which were based on medical records and the SmartCare system. SmartCare (31) is the national EHR system for HIV deployed by the Zambian Ministry of Health in collaboration with the U.S. Centers for Disease Control and Prevention (CDC).

### 2.2 Biosample analyses

Blood and urine samples were collected on the day of recruitment and at 2 weeks (+/-3 days) of treatment and only a blood sample was collected after 3 months (+/-1 month) of treatment. Approximately 4 mL of blood was collected in vacutainers containing ethylenediaminetetraacetic acid (EDTA) and potassium oxalate, and in plain vacutainers. A sterile container was used for the collection of approximately 12 mL of urine. CD4+ analysis was conducted within 2 hours on whole blood while the remaining blood was centrifuged, and the plasma or serum was stored at 2 ^0^C until testing within 72 hours of collection. Urine was stored at -80 ^0^C for less than 7 days until biomarker testing. The laboratory tests conducted on stored samples included viral load, serum and urine creatinine, serum sodium, serum potassium, serum and urine phosphate, blood urea nitrogen, serum glucose, and urine albumin. Urine KIM-1 and NGAL were analyzed using a commercial enzyme-linked immunosorbent assay (ELISA) from Elabscience (Wuhan, China) with reagent codes for human KIM-1 (E-EL-H0186) and human NGAL (E-EL-H0096). The calibrators, controls, and the samples for KIM-1 and NGAL were measured in duplicate and then the mean of the two measurements was used as the final concentration. The coefficient of variation for both biomarkers was less than 2%. The CD4+ cell count was analyzed using a Becton Dickinson (BD) FACS Calibur (BD. Biosciences, Erembodegem, Belgium), and viral load was analyzed using the COBAS*1* Ampliprep/COBAS*1* Taqman 48 HIV-1 Tests version 2 (Roche Diagnostics Corporation, Indianapolis, Indiana, USA) and a Hologic Panther (Hologic, Massachusetts, USA). The remaining tests were conducted on a Beckman Coulter AU480 chemistry analyzer (Beckman Coulter, Midrand, South Africa).

### 2.3 Data management and analysis

Study data were exported from REDCap to Stata 15 (StataCorp LCC., College Station, Texas, USA) for analysis (28, 29). Categorical variables were summarized using frequencies and percentages. The difference in proportions was computed using Pearson’s chi-square test or Fischer’s exact test. The normality of distribution for continuous variables was tested using the Shapiro–Wilk test. Median and interquartile ranges summarized the continuous variables. The difference between the two medians was tested by the Wilcoxon rank-sum test. The likelihood ratio test was used to test the assumption that introducing the two biomarkers (δKIM-1 and δNGAL) into the constrained model changed the prediction of nephrotoxicity. The baseline predictors fitted in the prognostic model included age, sex, BMI, urine albumin-to-creatinine ratio, CD4+ count, mean arterial pressure (MAP), viremia, serum creatinine, eGFR, and the biomarkers δKIM-1 and δNGAL. Data reduction was conducted using subject knowledge from the literature search, and hierarchical variable clustering eliminated collinearity and redundant variables. The Spearman index was used to select age, sex, BMI, baseline eGFR, baseline serum creatinine, mean arterial pressure, CD4+ count, viremia (HIV viral load > 1000 copies/mL), aviremia (HIV viral load >50 copies/mL but <1000 copies/mL), and the log-transformed urine albumin-to-creatinine ratio as covariates with predictive potential which were included in the final model. The logistic regression model was used to determine the association between the baseline predictors and TDF-associated nephrotoxicity. The assumption of linearity, heteroskedasticity, and association were tested. The model’s chi-square likelihood ratio was used to determine discrimination. The area under the curve (AUC) was drawn to determine the accuracy (linear prediction) of the model score and the sensitivity and specificity of the model were determined. Since logistic regression is based on mean least squares, outliers that can influence the ability of the model to accurately predict were eliminated by determining participants with high influential patterns [delta beta (dbeta) influence statistic > 0.2 and delta chi-square influence statistics (dx2) > 4].

A study of PLWH with viremia (cases) and aviremia (controls) with one control(s) per case was designed. Prior data indicated that the KIM-1 concentration increases by 0.20 in aviremia after 1 year of TDF use (21). If the true increase in KIM-1 in viremia is 0.43, we needed to study a minimum of 81 persons initiating TDF with viremia and 81 persons with aviremia to be able to reject the null hypothesis that the change in KIM-1 between persons with viremia and aviremia is equal with 80% power and α of 0.05 with a 10% adjustment for loss to follow-up. The continuity-corrected chi-squared statistic or Fisher’s exact test formula was used.

### 2.4 Ethics approval and consent to participate

The University of Zambia Biomedical Research Committee (UNZABREC) reviewed and granted ethical approval (Ref: 043-08-18) and the National Health Research Authority (NHRA) also granted permission to conduct the study.

## 3 Results

A total of 205 participants were studied of which two were not followed because they declined to provide blood and urine samples even though their demographics were collected, and the participants’ subsequent kidney outcomes were imputed in the analyses using Multiple Imputations by Chained Monte Carlo Equations (MICE). More details about the analyses can be found in our previous publication (32). Out of a total of 205 participants and 638 person-months, 45 (22%) developed nephrotoxicity during follow-up for an incidence rate of 70 cases per 1,000 person-months. The baseline clinical and laboratory characteristics of the participants stratified by TDF-associated nephrotoxicity are shown in **Table 1**. There were no differences in nephrotoxicity by sex, cigarette smoking, alcohol use, herb intake, viremia, baseline eGFR, or baseline serum creatinine between those who did and did not develop nephrotoxicity. None of the participants self-reported or had a documented history of comorbidities including diabetes mellitus, kidney disease, chronic diarrhea, hypertension, hepatitis B, hepatitis C, and tuberculosis. Furthermore, there were no statistically significant differences between the two groups of participants in median age, sex-stratified BMI, mean arterial pressure (MAP), duration of follow-up, viral load, CD4+ count, serum creatinine, fasting blood glucose (FBG), eGFR, serum cholesterol, urine creatinine, urine albumin-to-creatinine ratio, Kidney Disease: Improving Global Outcomes (KDIGO) stage of kidney dysfunction, and urine phosphate-to-creatinine ratio (**Table 1**).

**Table 1:** Comparison of baseline clinical and laboratory characteristics stratified by TDF-associated nephrotoxicity.

Variable	No Nephrotoxicity (N=158, 77.8%)	Nephrotoxicity (N=45, 22.2%)
Clinical, frequency (%)		
Sex [Female]*	89 (56)	31 (69)
Tobacco use	10 (8)	3 (9)
Alcohol use	52 (40)	11 (32)
Herbal medication/remedy use	25 (19)	8 (24)
Viremia	128 (81)	37 (82)
Baseline eGFR< 60 mL/min/1.73m2	4 (3)	2 (4)
KDIGO- G1 (eGFR≥ 90) G2 (eGFR> 60-89) G3a (eGFR> 45-59) G3b (eGFR> 30-44) G4 (eGFR> 15-29) G5 (eGFR< 15)	135 (85) 19 (12) 1 (0.6) 1 (0.6) 2 (1.3) 0	35 (78) 8 (18) 0 0 1 (2) 1 (2)
Median (IQR)		
Age [years]	36 (34, 37)	37 (32, 42)
Female BMI [kg/m^2^]	24 (21, 27)	24 (19, 27)
Male BMI [kg/m^2^]	22 (20, 25)	22 (18, 25)
Mean arterial pressure [mmHg]	98 (94, 102)	99 (93, 105)
Follow-up post-TDF [months]	3.5 (2.6, 4)	3.1 (2.3, 4)
Laboratory, median (IQR)		
Viral load [copies/mL]	62163 (6939, 262543)	64857 (15354, 191603)
CD4 cell count [cells/L]	230 (135, 454)	304 (166, 441)
Serum creatinine [µmol/L]	65 (53, 77)	56 (43, 77)
eGFR [mL/min/1.73m^2^]	132 (108, 143)	140 (105, 153)
Cholesterol [mmol/L]	4.2 (3.3, 5)	3.7 (2.7, 4.6)
Fasting blood glucose [mmol/L]	4.5 (4.2, 4.8)	4.2 (3.9, 4.5)
Urine creatinine [mmol/L]	9.7 (6.1, 15.4)	8.7 (6.6, 13.5)
Albumin-to-creatinine [mg/g]	103 (50, 263)	153 (61, 351)
Phosp-to-creatinine [mol/mol]	1.7 (1.4, 2)	2.1 (1.3, 3)
KIM-1_1-to-creatinine [pg/mg]	0.07 (0.04, 0.19)	0.07(0.04, 0.21)
KIM-1_2-to-creatinine [pg/mg]	0.14 (0.05, 0.27)	0.12 (0.05, 0.29)
NGAL_1-to-creatinine[pg/mg]	0.68 (0.43, 1.13)	0.70 (0.45, 1.15)
NGAL_2-to-creatinine [pg/mg]	0.60 (0.36, 1.03)	0.62 (0.36, 1.06)
δNGAL [pg/mg]	-0.02 (-0.23, 0.12)	-0.04 (-0.27, 0.12)
δKIM-1 [pg/mg]	0.01 (-0.04, 0.18)	-0.03 (-0.01, 0.16)

Viremia (HIV-1 RNA≥ 1000 copies/mL); KIM-1_1/2, kidney injury molecule-1 at baseline and 2 weeks; NGAL1/2, neutrophil gelatinase-associated lipocalin at baseline and 2 weeks; δNGAL & δKIM-1, change from baseline; KDIGO, Kidney Disease: Improving Global Outcomes.

Upon eliminating the four participants with high influential patterns (dbeta influence statistic > 0.2 and dx2 > 4) the model showed that every pg/mg increase in the difference between the baseline and 2-week concentration of urine KIM-1 (δKIM-1) was associated with a probability of 58% (95%CI 51, 69%) to develop TDF-associated nephrotoxicity. Furthermore, every 10 µmol/L increase in the baseline serum creatinine (creatinine_10pt) was associated with a 66% (95%CI 57, 74%) increase in the probability of developing TDF-associated nephrotoxicity. Furthermore, every 10 mL/min/1.73m^2^ increase in the baseline eGFR (egfr_10pt) was associated with a 74% (95%CI 63, 83%) increase in the probability of developing TDF-associated nephrotoxicity while every 1-year increase in age of the participant was associated with a 53% (95%CI 52, 55%) increase in the probability of developing TDF-associated nephrotoxicity. Every kg/m^2^ increase in BMI was associated with a 46% (95%CI 43, 49%) decrease in the probability of developing TDF-associated nephrotoxicity. Finally, compared to men, being a woman was associated with a 92% (95%CI 77, 98%) increase in the probability of developing TDF-associated nephrotoxicity (**Table 2**). However, baseline MAP, urine albumin-to-creatinine ratio (lnuacr1), CD4 (CD4_100pt), and viremia were not associated with a risk of nephrotoxicity (**Table 2**).

**Table 2:** Logistic regression model for the risk of TDF-associated nephrotoxicity.

Nephrotoxicity	Odds ratio (95% CI)	p-value	Probability (P) % (95% CI)
Creatinine_10	1.91 (1.31, 2.84)	**0.001**	66 (57, 74)
eGFR_10	2.91 (1.70, 5.02)	**0.000**	74 (63, 83)
BMI	0.87 (0.75, 0.98)	**0.036**	46 (43, 49)
MAP	1.01 (0.98, 1.04)	0.520	50 (49, 51)
*ln*uacr1	1.01 (0.77, 1.30)	0.964	50 (44, 57)
Age	1.14 (1.07, 1.22)	**0.000**	53 (52, 55)
CD4_100	1.04 (0.92, 1.18)	0.503	51 (48, 54)
Viremia	1.24 (0.37, 4.09)	0.350	55 (27, 80)
δKIM-1	1.41 (1.05, 2.26)	**0.002**	58 (51, 69)
Female	11.6 (3.42, 39.32)	**0.000**	92 (77, 98)

Creatinine_10, baseline serum creatinine/10; eGFR_10, baseline eGFR/10; BMI, baseline body mass index; MAP, baseline mean arterial pressure; CD4_100, baseline CD4 count/100; *ln*uacr1, log-transformed baseline urine albumin-to-creatinine ratio; viremia, viral load ≥ 80 copies/mL; δKIM-1, change in KIM-1 after 2 weeks of TDF use; probability (P) was calculated as P= OR/1+OR. The bold values mean there is a significant association P<0.05.

Fitting the model with δKIM-1 and δNGAL showed an improvement compared to another model we published previously (32) which did not have the biomarkers; a statistically significant improvement in the Akaike Information Criterion (AIC) was observed, p= 0.001. However, when the biomarkers δKIM-1 and δNGAL were introduced into the nested model one at a time, the improvement in model showed that only δKIM-1 improved the model, p= 0.001; δNGAL showed no significant change to the AIC, p= 0.15.

### 3.1 Model diagnostics and performance

The Hosmer–Lemeshow goodness-of-fit test for model calibration resulted in p = 0.16, suggesting that the data fitted well to the model. The model’s c-statistic obtained from the AUC resulted in a model discrimination of 0.81. Testing the model classification resulted in a positive predictive value (PPV) of 69% and a negative predictive value (NPV) of 85%. The sensitivity of the model was 30% while the specificity was 97%. Furthermore, the model correctly classified 84% of the participants.

### 3.2 Sensitivity analysis for urine KIM-1-to-creatinine for the probability of nephrotoxicity

**Figure 1:**
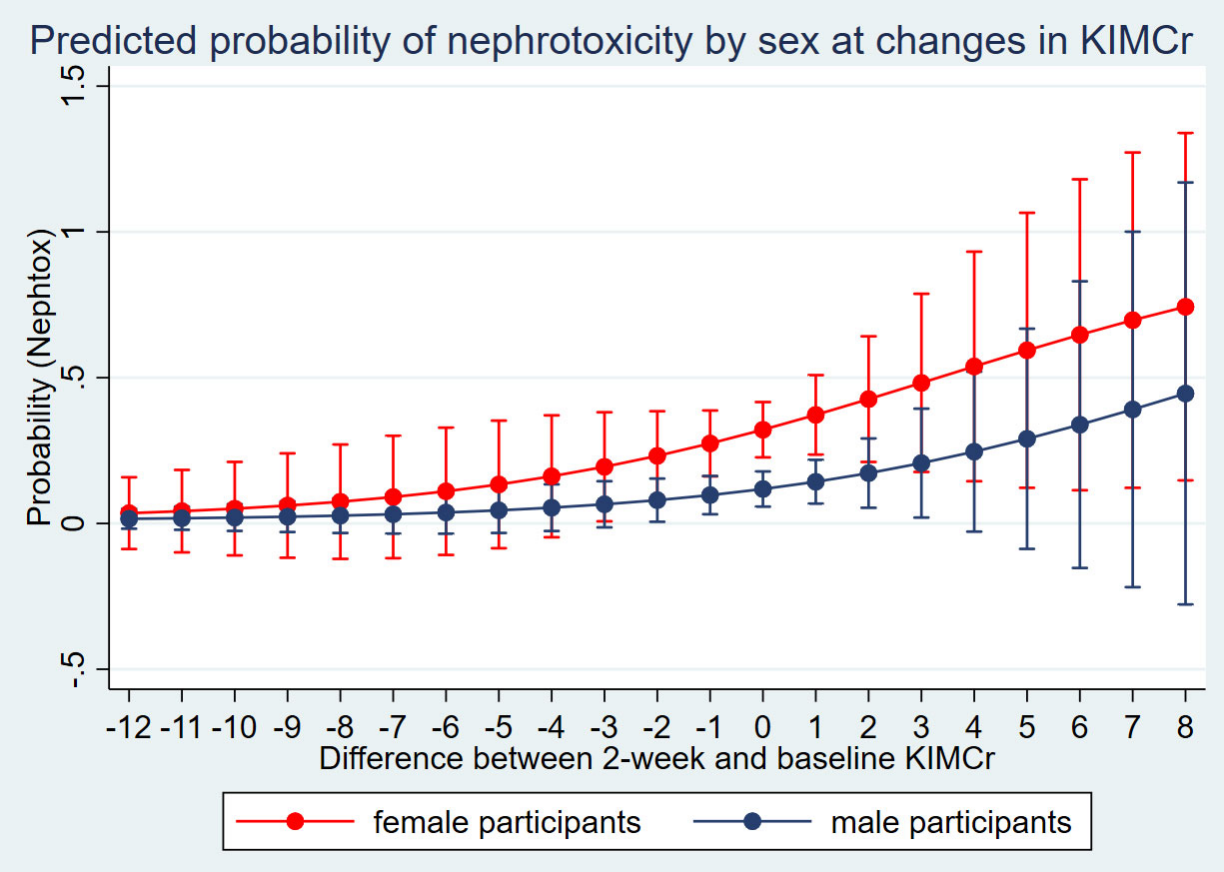
Sensitivity analysis of urine KIM-1 to predict the probability of nephrotoxicity by sex.

Further analysis of the 45 participants who developed nephrotoxicity based on our criteria showed that 15 (33%) had an eGFR reduction below 60mL/min/1.73, 32 (71%) had an eGFR decrease of 35%, while 36 (80%) had a serum creatinine increase of 50% from baseline; implying that some met more than one criterion. Analysis of the sensitivity of a unit change in the 2-week urine KIM-1-to-creatinine to predict the probability of nephrotoxicity amongst men and women showed higher marginal probabilities amongst women (**Figure 1**).

## 4 Discussion

In a cohort of adults living with HIV in Zambia with no or minimal kidney dysfunction at the start of TDF-based ART according to standard treatment initiation criteria, we measured the incidence of TDF-associated nephrotoxicity within 3 months and sought to identify the associated urine, blood biomarker, and demographic factors. We found that the baseline serum creatinine, eGFR, BMI, age, and change in urine KIM-1 within 2 weeks of TDF initiation predicted the risk of TDF-associated nephrotoxicity, which built upon evidence from previous studies. These data support the use of change in the concentration of KIM-1 within 2 weeks of initiating a TDF-based regimen to predict subclinical TDF-associated nephrotoxicity before overt manifestation at 3 months and could be used in clinical care to stratify the patients’ risk before initiating treatment with TDF. This could be invaluable, especially in low- and middle-income countries (LMICs) where there are no readily available options for treating end-stage kidney dysfunction such as chronic dialysis and kidney transplant which may result from concomitant TDF-associated nephrotoxicity.

A plethora of studies have linked various factors to the risk of TDF-associated nephrotoxicity including female sex, advanced age, pre-existing kidney insufficiency, unsuppressed HIV viral load, protease inhibitor-containing regimens (32–34), co-infection with hepatitis B or C, concomitant TDF use, comorbidities such as hypertension and diabetes, and advanced HIV clinical stage (WHO stages 3 or 4) (35–37). Our previous study (32) showed that treatment with a TDF-based regimen caused nephrotoxicity that manifested within the first 4 months among PLWH who initiated treatment with intact/preserved kidney function. There is no prognostic model for Zambia and most LMICs to estimate the risk of PLWH developing TDF-associated nephrotoxicity if treated. Consequently, anecdotal clinical/laboratory assessments are used to decide who initiates TDF-based therapy. The ability of these assessments to stratify patients by risk of nephrotoxicity is questionable considering the high cases of kidney disease reported in this population (32). Our study derived a prognostic model using routine clinical/laboratory data from patients at TDF initiation and discovered that no significant difference existed between the clinical/laboratory characteristics of those who developed kidney injury and those who did not. Finding no significant difference in the clinical/laboratory characteristics on which the current treatment algorithm is based [initiating TDF for PLWH with eGFR> 60 mL/min/1.73m^2^ (38)] exposed the futility of these assessments which are not based on found evidence. This algorithm based on clinical/laboratory assessments is pointless for kidney injury risk stratification as, for instance, it misclassifies approximately 87% of patients at risk of TDF-associated nephrotoxicity as being safe (32).

The primary aim of this study was to determine if including the change in the concentration of two biomarkers (KIM-1 and NGAL) within 2 weeks of initiating TDF-based therapy can predict subclinical nephrotoxicity before overt manifestation. The findings in this study showed that an increase in the difference between the baseline and the 2-week concentration of KIM-1-to-creatinine was associated with an increased probability of TDF-associated nephrotoxicity. This finding concurred with reports elsewhere that KIM-1 levels can be an early signal for kidney injury (19, 21, 25, 39). However, our finding that KIM-1 detected subclinical injury within 2 weeks of starting treatment with TDF was new considering that many studies measured the biomarker much later in the course of treatment, usually at a time when it has stabilized (18, 21, 40, 41). Furthermore, this study found that NGAL does not detect subclinical injury within 2 weeks of TDF use; this was also unique compared to reports by others that claimed NGAL could be used for the early detection of proximal tubular injury (42–44) but agreed with findings elsewhere (21) where the biomarker was assessed after 1 year of TDF therapy. The differences between our findings and other reports may be due to different times of assessing the change in the biomarker with our study assessing the change earlier in the treatment course.

The key findings of this study are that KIM-1 can predict subclinical TDF-associated nephrotoxicity within 2 weeks of initiating treatment for HIV infection while NGAL cannot. This suggests that using KIM-1 may augment risk stratification to prevent TDF-associated nephrotoxicity, especially among those predicted to have low/borderline risk of nephrotoxicity using the prognostic model we published previously (32). It should be underscored that most LMICs, such as Zambia, do not have options such as chronic dialysis and kidney transplant to effectively treat the ESKD that may result from TDF-associated nephrotoxicity, therefore, investing time in deriving and validating prognostic models that can predict and effectively discriminate patients by risk is crucial. Given that studies have linked nephrotoxicity to many kidney-related (45–49) and non-kidney-related complications (11, 48, 50–52) and that victims of kidney injury remain at high risk of complications even after removing the cause of injury (8, 9, 11), the findings of this study are important.

Finally, consistent with other reports (13, 32, 53, 54), this study also found that the baseline serum creatinine, eGFR, BMI, age, and being female were associated with the risk of developing nephrotoxicity. Advancing age increased the risk of TDF-associated nephrotoxicity, concurring with reports that an aging kidney suffers nephrosclerosis, which disturbs the transcriptomics, hemodynamics, and physiological behavior of the kidney, thereby impairing its response to injury (55, 56). The finding that women compared to men have a higher probability of developing TDF-associated nephrotoxicity is consistent with findings from South Africa and the USA (33, 57, 58); however, the pathophysiology of this difference remains unclear. Therefore, close monitoring of patients is crucial regardless of sex to mitigate the risk of kidney complications. Furthermore, on the one hand, an increase in serum creatinine (59, 60) was associated with an increased risk of TDF-associated nephrotoxicity consistent with findings elsewhere which showed that having pre-existing kidney impairment (61) increased the risk of TDF-associated nephrotoxicity (62, 63). On the other hand, paradoxically, an increase in baseline eGFR, which corresponds with better kidney function at baseline, seemed to be associated with a higher risk of TDF-associated nephrotoxicity (64, 65). This could be due to most participants initiating TDF with preserved kidney function entailing lower serum creatinine and high baseline eGFR with medians of 132 mL/min/1.73m2 and 140 mL/min/1.73m2, respectively. Extremely high and extremely low values are prone to regression to the mean. Additionally, since TDF typically significantly reduces kidney function within the first 3-6 months of therapy and the AKD criteria flagged nephrotoxicity when there is a 50% increase in creatinine, a 35% decrease in eGFR, or eGFR goes below 60mL/min/1.73m^2^, it is more likely that those with an elevated baseline eGFR level fulfilled any of the above AKD criteria than those around the mean. This study also found that an increase in BMI was inversely associated with the risk of TDF-associated nephrotoxicity. This was contrary to other studies (66, 67) that found a direct association. This was probably because our study was conducted among PLWH who are more likely to have a low BMI.

The strengths of this study were that it leveraged routine clinical biomarkers that are done among PLWH and included an enzyme-linked immunosorbent assay-based biomarker which is not difficult to test for and can be determined within 3 hours to improve the prediction of subclinical TDF-associated nephrotoxicity. Furthermore, these findings are unique because to our knowledge, no study has attempted to assess the changes in these biomarkers following short-term exposure to TDF and determined how this would forecast the risk of adverse kidney outcomes downstream. The limitation of this study is that the prognostic model needs external validation before use and might need optimization for other excluded populations such as children and adolescents.

## 5 Conclusions

In conclusion, the change in the KIM-1-to-creatinine ratio that follows 2 weeks of initiating treatment with TDF-based therapy can predict subclinical nephrotoxicity before overt manifestation 4 months later as TDF-associated nephrotoxicity. Using a prognostic model is a realistic and cost-effective way of preventing TDF-associated nephrotoxicity among PLWH, and these results build upon our previous study (32) that built a model using routine clinical and laboratory data; including kidney injury sensitive biomarkers to improve prediction was the next realistic step. One may worry about the cost implication and complexity of including such biomarkers, however, KIM-1 is a convenient ELISA-based biomarker that can be tested within 3 hours for approximately $10 per test. Considering the huge cost of treating ESKD and the threat to life it poses, including such a biomarker may be more cost-effective. However, it must be emphasized that there is a need to externally validate these prognostic models before integration into clinical practice.

## Data Availability

The original contributions presented in the study are included in the article/supplementary materials, further inquiries can be directed to the corresponding author/s.
